# Effect of Drying Whole and Half Chili Pods Using a Solar Dryer with CaCl_2_ Desiccant on Quality of Powder Chili

**DOI:** 10.1155/2021/9731727

**Published:** 2021-10-13

**Authors:** Nauas D. M. Romauli, Himsar Ambarita, Al Qadry, Hendrik V. Sihombing

**Affiliations:** ^1^Department of Food Science and Technology, Faculty of Agriculture, Universitas Sumatera Utara, Medan 20155, Indonesia; ^2^Department of Mechanical Engineering, Faculty of Engineering, Universitas Sumatera Utara, Medan 20155, Indonesia

## Abstract

This study investigated the quality of dried whole and half chili pods' powder dried with solar drying combined with CaCl_2_ desiccant compared with natural convection solar drying to produce the final chili powder product. Besides the sensory analysis, the quality parameters such as beta-carotene, moisture, vitamin C, and ash content were also observed. The results showed that 57 hours of drying time of whole and half chili pods using solar drying can reach moisture content below 5%. Beta-carotene, ash, and vitamin C contents of the whole chili were higher than those of half chili pods' dried pepper powder; they were20.38 ± 0.22 mg/100 g, 5.81 ± 0.15%, and 23.99 ± 0.57 mg/100 g, respectively. This study can guide the red chili drying process based on the quality of the dried chili powder.

## 1. Introduction

In 2019, Indonesia produced around 1,214,419 tons of chili, which increased 0.64% compared with the data in 2018, approximately 1,206,737 tons of chili [1]. The potency of Indonesia as a tropical country with a long duration of radiation, about 10-12 hours per day, was very highly potent to collect solar radiation by the solar collector, and the solar collector converted it into solar drier energy [2]. The performances of solar radiation have been tested to drying agricultural products such as cocoa beans and coffee beans [3–5]. Commonly, agricultural products such as red chili do the natural drying process utilizing sunlight with low operating costs as an effort to extend its shelf life due to its water content.

Red chili (*Capsicum annuum*), a member of the Solanaceae family, is well known as a nonclimacteric fruit, is the widely used spice in the world, and has an important biological activity of its phytochemicals as functional food which can provide health benefits, such as ascorbic acid or carotenoids [6–8]. As the product of secondary metabolism, vitamin C is an essential bioactive compound that can prevent several diseases [9]. Beta-carotene, a precursor of vitamin A and as one of the main carotenoids, provides red color in ripe red chili. Oxidation of vitamin A can be stimulated by heat or light, which can cause the loss of color and decrease vitamin A's activity [10]. Natural drying takes a longer drying time process to reduce the red chili moisture content to below 11% [11]. The drying process using high heat to evaporate the moisture contained in the material can causes changes in color, aroma, and vitamin content [12].

The solar dryer from solar energy systems is more promising than natural convection solar drying methods due to more hygienic characteristics and shorter drying times. The first objective of this study was to study the performance of indirect solar drying combined with CaCl_2_ desiccant to dried red chili. Another aim was to determine the effects of the whole chili and half chili pods dried with solar drying compared with natural convection solar drying on the quality of chili powder.

## 2. Materials and Methods

### 2.1. Sample Preparations

Good quality of fresh fully ripe red chili with height 13-17 centimeters long and weight 4-6 grams per fruit range of size was purchased from a local market in the Karo Regency. The red chili was washed and sorted, and stems were removed. Whole or cut-in-half red chili pods were spread in a single layer on a stainless steel wire tray for natural convection solar drying or solar drying.

### 2.2. Preparation of Solar Drying and Processing of Dried Red Chili

The solar dryer was operated on the rooftop of the Sustainable Research Centre of Energy, Universitas Sumatera Utara. During the experimental period, solar radiation and ambient temperature were measured by a HOBO Micro Station Data Logger (version 3.7.12). Temperature, humidity, and mass of red chili inside the drying box or solar collector were measured using sensor DHT22, type J thermocouples, and a load cell weight system data logger with an Agilent type 3497A data acquisition system to record all the measurements. 1 kg of red chili for the experimental treatments was spread on each of the stainless steel trays inside a closed drying box of solar drying and on the stainless steel trays dried directly in the open sun for the natural convection solar drying. Red chili was dried starting at 9:00 AM until 6:00 PM. For the solar drying experiment using desiccant during nighttime, desiccant CaCl_2_ was added inside the drying box, and the drying chamber was closed until the next morning. Drying time ended around three days when the dried chili from inside the solar drying box reached a constant weight, and then we ground all the dried chili into a powder.

**Figure 1:**
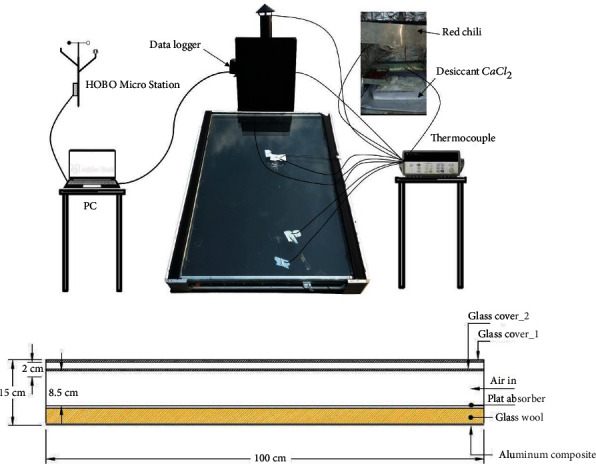
The solar dryer details from solar energy systems.

The solar dryer details in this study are designed as follows (see Figure 1) with the solar collector specification as shown in Table 1.

### 2.3. Experimental Design and Statistical Analysis

A2 × 3factorial design with experimental factors using two different forms of red chili (whole or half chili pods dried with three types of drying methods (solar dryer combined with CaCl_2_ desiccant, solar dryer without CaCl_2_ desiccant, and natural convection solar drying)) was analyzed statistically to ANOVA using the statistical software SPSS. All the data were carried out in triple replications and reported as mean value ± SD (standard deviations).

### 2.4. Chemical Analysis and Sensory Analysis

The beta-carotene, moisture content, vitamin C, and ash content of the final product were analyzed. The AOAC method [13] determines the moisture content and ash content in a muffle furnace. Color values were quantified using *L*∗, *a*∗, and *b*∗ parameters by using Chroma Meter CR-400. Total color change (Δ*E*∗) was calculated using the formula Δ*E*∗ = [(Δ*L*∗)^2^ + (Δ*a*∗)^2^ + (Δ*b*∗)^2^]^1/2^ [14]. Vitamin C was measured by UV-Vis spectrophotometry at a wavelength of 518 nm [15]. The beta-carotene was determined using a spectrophotometer at 452 nm [16]. Sensory analysis in chili powder was conducted monadically by 60 untrained panelists using the 7-point hedonic scale with 1, 4, and 7 for “strongly dislike,” “indifferent,” and “strongly liked,” respectively, for color, aroma, texture, and overall acceptability [17].

## 3. Results and Discussions

### 3.1. Performance of Solar Drying Combined with CaCl_2_ Desiccant

**Figure 2:**
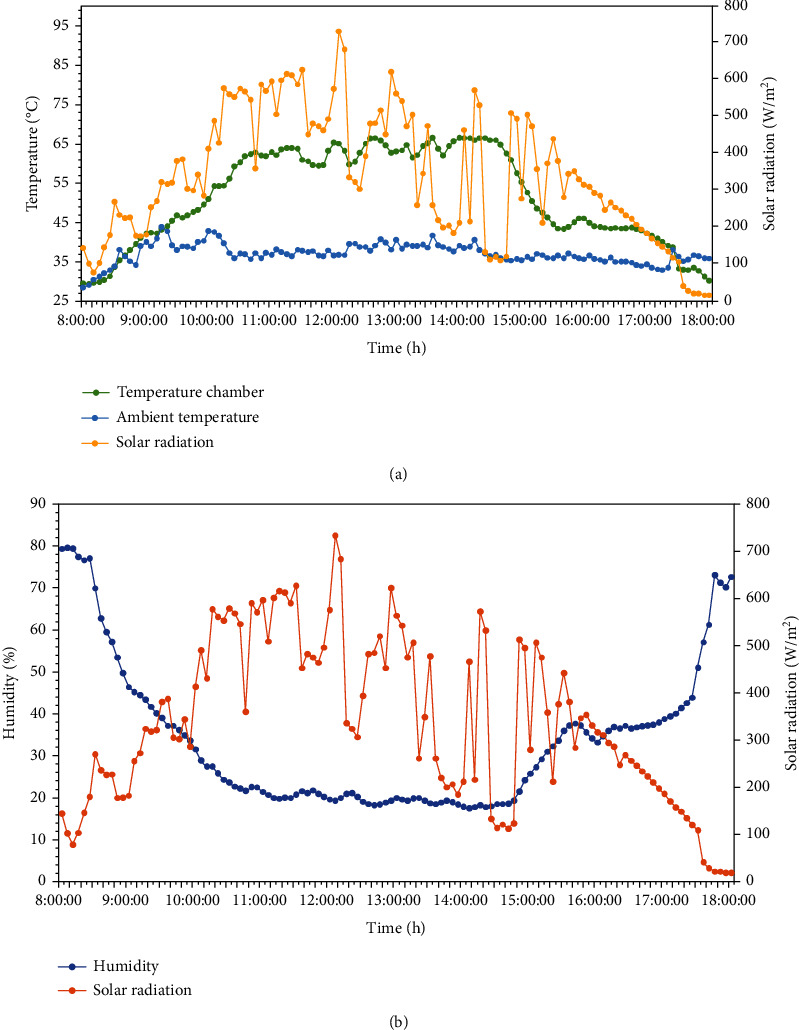
(a) Temperature inside the solar drying box at daytime. (b) Relative humidity inside the solar drying box at daytime.

The experimental results in Figure 2 show that temperature within the solar dryer at 8:00 AM increased from 29.4°C ± 0.36 to 63.53°C ± 5.92 at 2:15 PM and decreased to 35.1°C ± 1.32 at 6:00 PM with relative humidity of the solar dryer ranging from 22.9% ± 12.1 to 78.1% ± 4.14, respectively. As the solar radiation increased, the temperature inside the dryer also increased. The maximum temperature inside the drying box during drying time was around 56.7°C to 66.6°C. It was higher by 14°C to 22°C than the ambient temperature (the ambient temperature was around 42.1°C to 43.8°C). The temperature inside the solar dryer was higher than the ambient air temperature at daytime, which indicates an expectation for a higher rate of moisture removal in the solar dryer than natural convection solar drying.

**Figure 3:**
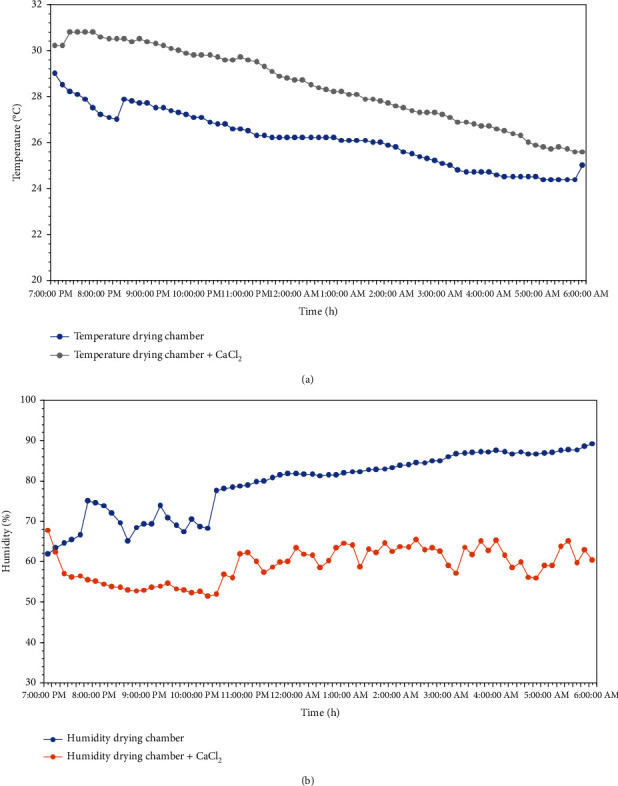
(a) Temperature inside the solar drying box at nighttime. (b) Relative humidity inside the solar drying box at nighttime.

At nighttime (Figure 3), the humidity inside the solar dryer with desiccant CaCl_2_ was lower, and the temperature was higher than the solar dryer without added desiccant. Desiccants can absorb moisture from the air and reduce humidity inside a sealed drying box. Low relative humidity may enhance the quick-drying process because it has more capacity to dry than that with higher humidity.

**Figure 4:**
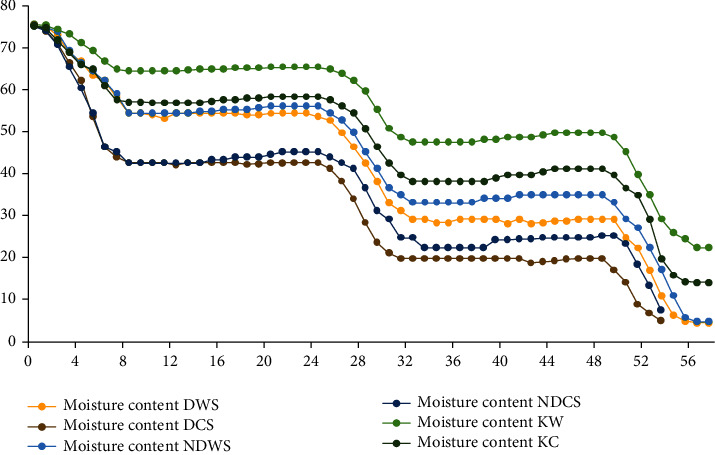
Moisture content (wb%) versus drying time (h). DWS: whole pods dried using desiccant in solar drying; NDWS: whole pods dried without using desiccant in solar drying; KW: whole pods dried using natural convection solar drying; DCS: half chili pods dried using desiccant in solar drying; NDCS: half chili pods without using desiccant in solar drying; KC: half chili pods dried using natural convection solar drying.

Figure 4 shows that 1 kg of red chili by using this solar dryer reduced the moisture content from near 80% (wet basis) to less than 5% (wet basis) around 57 hours of drying time (including nights). During the nighttime, the moisture content of NDWS and NDCS slightly increased. It was because the solar dryer without using desiccant inside the drying box at night has higher relative humidity, and the dried object will reabsorb the moisture. The moisture content of DWS and DCS is reduced on average 0.01 g/g, and the moisture content of the other dried samples slightly increased on average 0.3 g/g during nighttime. The drying rate was relatively high during the daytime, which corresponded to the period of high temperature inside the drying chamber and high solar radiation intensity to evaporate moisture from the drying materials over the drying process. Compared with the results obtained in the previous study with a slope angle around 60° and different material components (tin-Rockwool-Styrofoam-wood) and size of the solar collector (200 cm × 50 cm × 10 cm) [18–20], this experiment gave lower moisture content results.

In the drying process, the water at the surface of the red chili will evaporate, and also the water in the inner part of the red chili will migrate to the surface to disappear. The pretreatment method where red chili was cut in half before drying (DCS, NDCS, and KC) makes it easy for water in the inner part to migrate and leads to a shorter drying time than whole pods. Both DCS and NDCS have a quicker drying time, around 55.5 hours. DCS in the condition using desiccant at 52 hours of drying time gave the lowest moisture content. KC and KW using natural convection solar drying have the longest drying time between the three drying methods. A similar result was reported that natural convection solar drying has a low rate of moisture evaporation from chili surface to air, which caused an increase in drying time [21].

### 3.2. Beta-Carotene, Moisture Content, Vitamin C, and Ash Content in Chili Powder

The effect of pretreatment size (whole or half chili pods) on beta-carotene, moisture content, vitamin C, and ash content of dried chili powder is shown in Table 2. Capsicum fruits were rich in carotenoids, such as beta-cryptoxanthin, violaxanthin, beta-carotene, capsorubin, and capsanthin. Mainly, beta-carotene was the most abundant in red or yellow capsicum fruits [22]. Ascorbic acid in fresh capsicum fruits varies depending on cultivars and ripening stages. Generally, beta-carotene and vitamin C were susceptible during food processing and unstable at high temperatures [23–25]. The beta-carotene and vitamin C content of chili powder from dried half chili pods (DCS, NDCS, and KC) were lower than those of whole dried pods. The effect of the treatment form of red chili before the drying process demonstrated the most significant impact on vitamin content. Kim et al. [26] reported that according to the Food Composition Table, the ascorbic acid content of dried chili is about 26 mg/100 g. The content is slightly higher content than in our results, but that means our drying methods destroyed less ascorbic acid than using natural convection solar drying. Moisture content is important to ensure optimum quality and product safety [27]. According to Indonesian National Standard (SNI 01-3389-1994), the moisture content of dried red chili products has to be below 11% [28]. The moisture content was analyzed and determined to range from 4.31 to 4.88%. The moisture contents were not significantly (*p* ≤ 0.05) affected by the treatment form of red chili dried with solar drying using desiccant or without desiccant. Ash content was found to increase from 3.54 to 5.81% due to the removal of water, which increased the dry matter and ash values [29].

### 3.3. Color Value in Chili Powder

The color values between the whole and half chili pods using different drying methods are presented in Table 3. The *L*∗ value indicates the beam of light (darkness-whiteness), the *a*∗ value indicates the color between red to green, and the *b*∗ value indicates the color between yellow and blue for color vision to the human eye. The *a*∗ color value of dried chili powder of whole and half chili pods using natural convection solar drying was found to be significantly lower (*p* ≤ 0.05) than that dried in solar drying. The effect of using desiccant or without desiccant on the color qualities (*L*∗, *a*∗, and *b*∗) of dried whole chili pods was not significantly different among the samples (*p* ≤ 0.05). Drying with natural convection solar drying led to a decrease in lightness (*L*∗), redness (*a*∗), and yellowness (*b*∗) values in chili powders. The decrease in color value might be because of the carotenoid degradation and, as a consequence, may lead to a loss in coloring due to oxidation. The *L*∗, *a*∗, and *b*∗ values of dried half chili pods' powder (DCS and NDCS) are higher than those of dried whole chili pods due to the shorter drying time of dried half chili. Getahun et al. [30] reported that shortening the drying time minimizes the color degradations and gives high-quality attributes of dried red chili. Δ*E*∗ values between dried red chili samples and fresh red chili samples varied between +9.18 and +24.95. The lower value of the Δ*E*∗ means less difference in color between the two colors. Δ*E*∗ values between DWS and NDWS were in a similar range (*p* ≤ 0.05). The nearest color change (+9.18) was obtained in NDCS samples.

### 3.4. Sensory Analysis in Chili Powder

**Figure 5:**
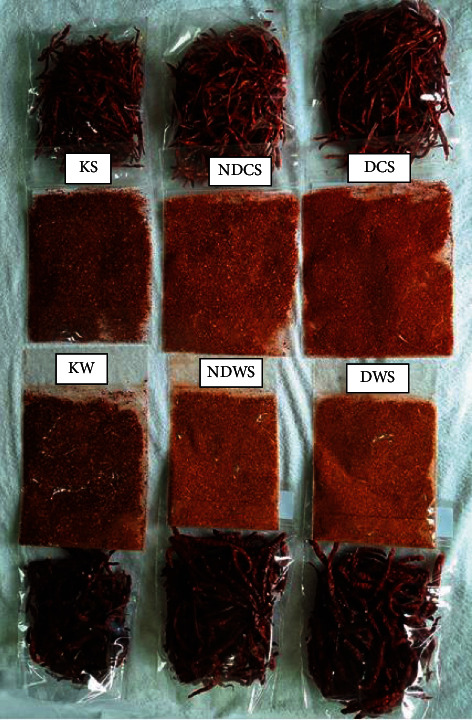
Comparison color of the dried chili product. DWS: whole pods dried using desiccant in solar drying; NDWS: whole pods dried without using desiccant in solar drying; KW: whole pods dried using natural convection solar drying; DCS: half chili pods dried using desiccant in solar drying; NDCS: half chili pods without using desiccant in solar drying; KC: half chili pods dried using natural convection solar drying.

Besides pungency, color is also the main characteristic of red chili that determines its quality. The surface color showed in Figure 5 is one of the critical evaluation parameters by the consumer at first glance due to the acceptance or rejection of the final product. It considers the carotenoid (yellow, orange, and red) pigment's destruction based on color. It will be related to the browning reaction that gave the dark color of the food surface [31].

The results in Table 4 show no significant difference (*p* > 0.05) in the value of texture and aroma. The sensory analysis of color attributes of whole chili pods dried in solar drying (with or without desiccant) was significantly different among the samples (*p* ≤ 0.05). The highest score for overall acceptability was observed from the whole chili sample dried in solar drying with or without desiccant, 5.95 and 5.9, respectively.

## 4. Conclusions

Solar drying with the ambient temperature around 42.1°C to 43.8°C took 57 hours to reduce the red chili moisture content from 76% to below 5%, while dried red chili power using natural convection solar drying still has higher moisture content (more than 11%). Although the drying time becomes shortened with the pretreatment of cutting in half the red chili before drying, it gave lower vitamin C and beta-carotene content of dried chili powder. Moisture contents were not significantly affected by treatment using desiccant or without desiccant on solar drying. For overall acceptability, the whole chili sample dried in solar drying with or without desiccant gave the highest score than conventional drying. The main conclusion is that drying the whole chili using indirect solar drying added with CaCl_2_ desiccant in the night can be an alternative way for rural farmers, where mechanical dryers gave high fuel costs for drying red chili. Further studies, such as modification of the solar collector, are needed to investigate.

## Figures and Tables

**Table 1:** Specification of the solar collector.

Parameter	Specifications
Drying box size	50 cm × 50 cm × 70 cm
Collector size	150 cm × 100 cm × 15 cm
Collector material component	Plat absorber-glass wool-aluminum composite
Slope angle	30°

**Table 2:** Beta-carotene, moisture content, vitamin C, and ash content of chili powder.

Sample	Beta-carotene (mg/100 g)	Moisture content (%)	Vitamin C (mg/100 g)	Ash (%)
Fresh red chili	40.10 ± 0.25^a^	76.56 ± 0.87^a^	83.16 ± 1.04^a^	1.28 ± 0.06^e^
DWS	20.38 ± 0.22^b^	4.63 ± 0.31^d^	23.99 ± 0.57^b^	5.81 + 0.15^a^
NDWS	17.06 ± 0.23^c^	4.88 ± 0.33^d^	23.96 ± 0.74^b^	5.32 ± 0.30^b^
KW	15.81 ± 0.57^d^	20.97 ± 0.78^b^	13.30 ± 0.52^d^	3.60 ± 0.25^d^
DCS	14.62 ± 0.41^e^	4.31 ± 0.07^d^	23.28 ± 0.54^bc^	5.07 ± 0.14^bc^
NDCS	14.43 ± 0.33^e^	4.53 ± 0.20^d^	23.01 ± 0.60^c^	4.98 ± 0.35^c^
KC	13.70 ± 0.12^f^	13.89 ± 1.06^c^	12.81 ± 0.59^d^	3.54 ± 0.31^d^

Mean ± SD values with different superscripts in a column are significantly different (*p* ≤ 0.05). DWS: whole pods dried using desiccant in solar drying; NDWS: whole pods dried without using desiccant in solar drying; KW: whole pods dried using natural convection solar drying; DCS: half chili pods dried using desiccant in solar drying; NDCS: half chili pods without using desiccant in solar drying; KC: half chili pods dried using natural convection solar drying.

**Table 3:** The color value of chili powder.

Color value	Fresh chili	DWS	NDWS	KW	DCS	NDCS	KC
*L*∗	31.40 ± 0.51^a^	25.68 ± 1.66^c^	25.38 ± 0.82^c^	22.85 ± 0.76^d^	28.83 ± 1.05^b^	30.06 ± 1.59^ab^	20.96 ± 0.81^e^
*a*∗	29.16 ± 0.23^a^	12.19 ± 1.19^b^	11.93 ± 1.26^b^	6.67 ± 0.52^e^	19.26 ± 0.83^b^	20.26 ± 0.68^b^	9.84 ± 1.47^d^
*b*∗	7.93 ± 0.55^b^	6.29 ± 1.45^c^	6.05 ± 0.76^c^	1.53 ± 1.21^d^	9.86 ± 0.80^a^	8.49 ± 0.34^b^	2.55 ± 0.70^d^
Δ*E*∗		+18.08 ± 1.35^c^	+18.39 ± 1.21^c^	+24.95 ± 0.64^a^	+10.54 ± 0.74^d^	+9.18 ± 0.59^e^	+22.62 ± 1.41^b^

Mean ± SD values with different superscripts in a column are significantly different (*p* ≤ 0.05). DWS: whole pods dried using desiccant in solar drying; NDWS: whole pods dried without using desiccant in solar drying; KW: whole pods dried using natural convection solar drying; DCS: half chili pods dried using desiccant in solar drying; NDCS: half chili pods without using desiccant in solar drying; KC: half chili pods dried using natural convection solar drying.

**Table 4:** Sensory analysis of dried chili powder.

Sensory attribute	DWS	NDWS	KW	DCS	NDCS	KC
Color	6.08 ± 0.10^a^	6.05 ± 0.10^a^	5.84 ± 0.10^b^	5.87 ± 0.14^b^	5.78 ± 0.02^b^	5.81 ± 0.04^b^
Texture	5.83 ± 0.21^a^	5.75 ± 0.21^a^	5.66 ± 0.04^a^	5.61 ± 0.16^a^	5.76 ± 0.34^a^	5.83 ± 0.22^a^
Aroma	5.77 ± 0.30^a^	5.75 ± 0.31^a^	5.76 ± 0.05^a^	5.68 ± 0.05^a^	5.85 ± 0.14^a^	5.85 ± 0.20^a^
Overall acceptability	5.95 ± 0.13^a^	5.9 ± 0.24^ab^	5.31 ± 0.08^d^	5.66 ± 0.13^bc^	5.71 ± 0.10^bc^	5.51 ± 0.10^cd^

Mean ± SD values with different superscripts in a column are significantly different (*p* ≤ 0.05). DWS: whole pods dried using desiccant in solar drying; NDWS: whole pods dried without using desiccant in solar drying; KW: whole pods dried using natural convection solar drying; DCS: half chili pods dried using desiccant in solar drying; NDCS: half chili pods without using desiccant in solar drying; KC: half chili pods dried using natural convection solar drying.

## Data Availability

The data used to support the findings of this study are included within the article.
